# Iatrogenic Blepharoptosis: Multimodal Management and Treatment Technique With Botulinum Toxin Type A

**DOI:** 10.1155/drp/8827594

**Published:** 2025-10-17

**Authors:** Rengifo-Palacios Jaime Alberto, Macías-Arias Paola Andrea, Uribe-Posada Maria Paulina, Lopera-Botero Luisa

**Affiliations:** ^1^Dermatology, Pontificia Bolivariana University, Medellín, Colombia; ^2^Dermatology, Federico Lleras Acosta Dermatological Hospital, Bogotá, Colombia

**Keywords:** blepharoptosis, botulinum toxin, eyelid, iatrogenic, ptosis

## Abstract

**Blepharoptosis as an Aesthetic Complication:**

Eyelid ptosis, or blepharoptosis, following esthetic treatment of the upper third with botulinum toxin Type A (BoNT-A) is a complication with a variable incidence depending on the injector's experience. Among unexperienced injectors, it ranges from 2.5% to 5.4% and approximately 0.51% to 1% in experienced injectors. Blepharoptosis is commonly defined as an eyelid located between 1.5 and 2 mm below the scleral-corneal limbus. It occurs because of the local spread of botulinum toxin, affecting the levator palpebrae superioris muscle, one of the principal muscles for elevating the superior eyelid. It typically becomes evident 3–14 days after BoNT-A application and resolves spontaneously after approximately 3 months, once the toxin's effect subsides. Even though it resolves with time, it can cause great distress for the patient and the physician.

**Treatment Modality:**

In turn, knowing the anatomy of the face in high detail will help the physician treat and prevent this complication, which can be avoided with correct training and application. Once it has happened, it is important to recognize the severity of the blepharoptosis (which is classified as mild, moderate, or severe), in order to decide whether to use oxymetazoline or apraclonidine eye drops, muscle exercises, vibrating devices, radiofrequency, and the latest option described with pretarsal BoNT-A application. Even though the treatment is challenging, and evidence is scarce, here we present a literature review and some clinical cases of successful treatment with pretarsal BoNT-A in iatrogenic blepharoptosis following esthetic treatment of the upper third.

**Objective:**

This review highlights the importance of facial anatomy knowledge to minimize potential complications associated with BoNT-A application. It also describes the clinical classification and management of iatrogenic blepharoptosis based on severity, with special emphasis on the pretarsal BoNT-A application technique.

**Methods of Literature Search:**

A literature search was conducted using electronic databases (PubMed, MEDLINE, Embase, and Google Scholar), focusing on upper third anatomy, prevention of iatrogenic blepharoptosis secondary to BoNT-A application, classification, and therapeutic options based on severity.

**Results:**

Iatrogenic eyelid ptosis after BoNT-A application results from the neurotoxin spreading to the levator palpebrae superioris muscle. Current therapeutic options include sympathomimetic eye drops, vibration therapy, facial exercises, radiofrequency, and pretarsal BoNT-A application. This review emphasizes anatomical knowledge, risk factors' identification, and anatomical landmarks to minimize complications. The pretarsal treatment technique for iatrogenic ptosis using BoNT-A is also detailed.

**Limitations:**

The limitations of this review consist of the number of patients, which is very limited; another limitation is that none of the patients had severe ptosis to prove the treatment.

**Conclusion:**

Blepharoptosis following esthetic BoNT-A treatment is a rare complication among trained injectors. Knowledge of therapeutic options, including pretarsal BoNT-A injection techniques, is crucial for managing this complication, which can have significant esthetic and functional impacts.

## 1. Introduction

Eyelid ptosis, or blepharoptosis, following esthetic treatment with botulinum toxin Type A (BoNT-A) in the upper third is a complication with variable incidence. It ranges from 2.5% to 5.4% among inexperienced injectors and approximately from 0.51% to 1% among expert injectors. This suggests that the complication decreases as the injector's learning curve improves. Furthermore, when it does occur, it is most often mild to moderate in severity [1–5].

This complication arises from the local dissemination of the neurotoxin from the periocular region to the levator palpebrae superioris muscle in the intraorbital compartment [1, 2]. It typically becomes evident 3–14 days after BoNT-A application and resolves spontaneously after approximately 3 months, once the toxin's effect subsides [2]. However, it can be a distressing event for both the patient and the injector. Therefore, minimizing its effects promptly is crucial due to the esthetic and visual discomfort caused by reduced eyelid opening [1].

Understanding the anatomy of the supraorbital region and orbital roof is essential for recognizing danger zones and serves as a key tool in preventing iatrogenic eyelid ptosis [2].

## 2. Anatomy

Anatomically, the support of the upper eyelid is provided by the tarsal plate, consisting of dense connective tissue attached medially to the anterior lacrimal crest of the maxilla bone through the medial palpebral ligament and laterally to the orbital tubercle of the zygomatic bone through the lateral palpebral ligament [6].

The tarsal plate is associated with the levator palpebrae superioris muscle, a triangular muscle originating from the posterior roof of the orbit just above the optic foramen. It inserts into the anterior surface of the tarsal plate, with some fibers directly attaching to the eyelid skin through the orbicularis oculi muscle. The posterior tendon of this muscle gradually thickens as it passes over the eyeball. It is innervated by the superior branch of the third cranial nerve (oculomotor nerve), which enters through the muscle's inferior surface. Blood supply is provided directly by the ophthalmic artery and indirectly by its supraorbital branch [6, 7].

The levator muscle functions by elongating its aponeurosis medially and laterally, elevating the eyelid. This action is counteracted by the orbicularis oculi muscle in its palpebral portion, which is linked to the superior rectus muscle by a ligament that elevates the eyelid during upward gaze. Conversely, when the eye closes, the orbicularis oculi contracts while the levator relaxes [7].

Another muscle responsible for eyelid elevation is the superior tarsal muscle (Müller's muscle), composed of smooth muscle fibers running along the inferior surface of the levator muscle to the apical edge of the tarsal plate. It is innervated by postganglionic sympathetic fibers from the superior cervical ganglion. This muscle contributes to eyelid elevation, particularly during emotional states such as fear or excitement, when sympathetic stimulation opens the eye further [6, 7]. Consequently, dysfunction in these muscles, either through direct muscle damage or loss of innervation, results in upper eyelid ptosis [6, 8].

The frontal region and the intraorbital compartment are separated by the fibrous attachments of the orbital septum at the superior orbital rim. While the orbital septum represents a true anatomical barrier, it has weak points that allow unintentional toxins to spread. These points are located around the superior neurovascular bundles: the supratrochlear, supraorbital, and lacrimal pedicles [2].

## 3. Blepharoptosis

The definition of blepharoptosis can vary depending on the literature. Ptosis is defined as an eyelid located between 1.5 and 2 mm below the scleral-corneal limbus. Other definitions include a superior marginal reflex distance below 2 mm, which is the distance between the corneal light reflex and the upper eyelid margin. In addition, an asymmetry of more than 2 mm between both eyes is considered ptosis [2].

It is important to note that the height of the palpebral fissure does not vary with age, although it can differ due to ethnicity and genetics, typically measuring around 11–12 mm ± 2 mm [9].

**Figure 1:**
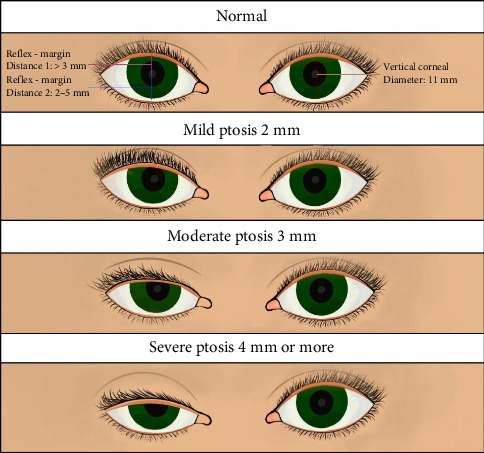
Blepharoptosis severity classification.

The severity of eyelid ptosis is evaluated based on the marginal reflex distance using the scale in Table 1 [10, 11] (Table 1 and Figure 1).

The etiology of eyelid ptosis is often multifactorial. Causes can be congenital or acquired. The causes of eyelid ptosis can be classified into the following categories: neurogenic, myogenic, aponeurotic, mechanical, and traumatic [10].

Neurogenic ptosis occurs due to abnormalities in the oculomotor or sympathetic nerve stimulus or the central nervous system, which can result in eyelid drooping; myogenic ptosis arises from dysfunction of the levator palpebrae superioris muscle, limiting its ability to elevate the eyelid; the aponeurotic ptosis, the most common type, results from the senescent detachment or weakening of the aponeurosis and palpebral tendon tissues, causing the eyelid to sag; and finally, the traumatic ptosis is due to partial or complete injury to the levator muscle, impairing its function [12, 13].

Ptosis resulting from the cosmetic application of BoNT-A is classified as myogenic, caused by impaired transmission of electrical impulses at the neuromuscular junction [2, 3].

BoNT-A works by blocking the release of acetylcholine, the neurotransmitter responsible for muscle contraction. This diminishes the muscle's ability to contract and maintain tension [14].

The risk factors for post-BoNT-A blepharoptosis can be grouped into three categories, patient-related factors, product-related, and injector-related [3].

The patient-related factors include young age, chronic sun damage, a short forehead, thick or inelastic skin, anatomical variations in the supraorbital foramen, recent facial surgery, a history of peripheral facial paralysis or prior ptosis, and neurological disorders such as myasthenia gravis or multiple sclerosis [3, 15].

For product-related factors, these include incorrect dilution or the use of lower-quality toxins [3, 15].

Finally, for injector-related factors, these include poor injection technique, excessive reconstitution or injection volumes, errors in anatomical site selection, inappropriate needle gauge, and injection speed [3, 15].

Paternostro et al. conducted a cadaveric injection study to evaluate the potential pathways through which the neurotoxin might spread into the intraorbital space, affecting the levator palpebrae superioris muscle. Their findings suggest that a low injection volume, superficial injections in the supraorbital area, and angling the needle tip away from the supratrochlear foramen (toward the contralateral temporal region) during corrugator injections can increase the safety profile of esthetic botulinum toxin treatments in the glabellar region [5].

Higher injection volumes increase the likelihood of toxin migration to adjacent regions. The supratrochlear and supraorbital neurovascular bundles are potential entry points into the intraorbital space, with the latter posing a higher risk due to its proximity to the levator palpebrae superioris muscle [5, 16].

According to the above, we emphasize on the preventive strategies described in the literature to minimize the risk of iatrogenic blepharoptosis.

In the first place, a focused medical history is essential to identify patient-related risk factors that may affect BoNT-A's efficacy or performance. It is critical to ask about prior injections and their outcomes, especially adverse effects. Photographic documentation in both static and dynamic states is recommended, as up to 90% of the population shows some degree of eyebrow asymmetry. Postprocedure care education is also crucial, especially the importance of avoiding massages in the treated area [2].

**Figure 2:**
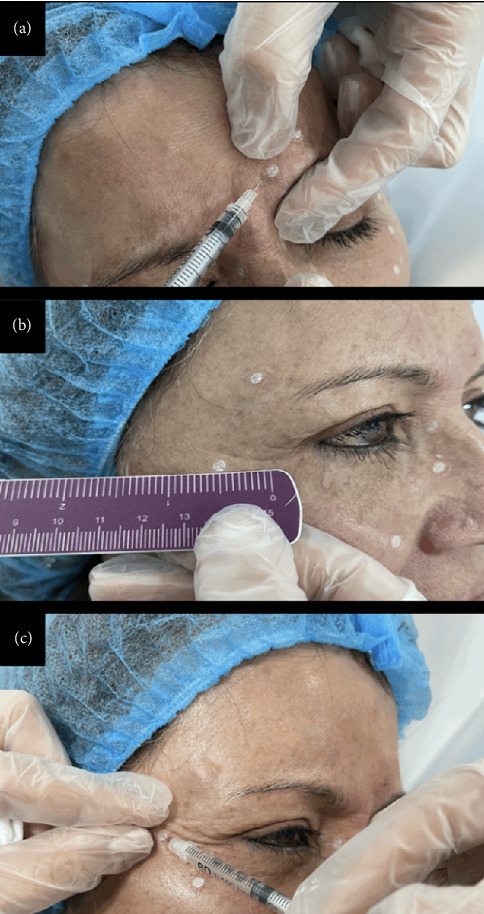
Special considerations in injection technique.

Regarding the strategies associated with the procedural technique, King et al. emphasize on the importance of specific anatomical considerations and injection precautions, such as, injecting at least 1 cm above the eyebrows when treating the glabellar region, applying digital pressure to the supraorbital rim while injecting the corrugator muscle, directing the injection superiorly and away from the orbit, maintaining digital pressure for 4–6 s after injection, using superficial injections in the supraorbital area, keeping a safety margin of 1 cm from the orbital rim and directing the injection laterally when treating the orbicularis area corresponding to wrinkles, avoiding injections directly beneath the eye in patients with significant scleral exposure or negative lid retraction tests, and lastly, considering the depth of the muscle to ensure appropriate injection technique [3, 5, 17] (Figures 2(a), 2(b), and 2(c)).

**Figure 3:**
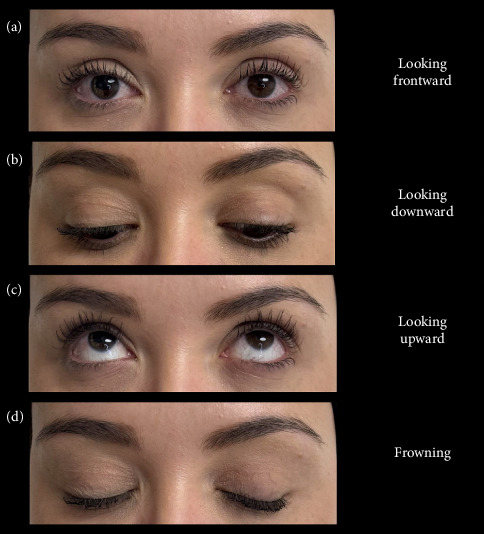
Eyelid physical examination.

During the clinical evaluation of eyelid opening height, the examiner should instruct the patient to look straight ahead and position their head with the chin slightly raised, avoiding any attempt to lift the eyelids [18]. Similarly, the evaluation should include observing the gaze straight ahead, upward, downward, and with the eyelids tightly closed (Figure 3).

Some treatment strategies for iatrogenic blepharoptosis include the administration of medications such as oxymetazoline hydrochloride, apraclonidine hydrochloride, or phenylephrine hydrochloride eye drops, as well as physical measures or pretarsal transdermal injections of BoNT-A [2].

The application of BoNT-A requires a transdermal injection technique into the pretarsal portion of the orbicularis oculi muscle. This portion of the muscle autonomously functions to close the eye during blinking. By reducing the baseline force in this region, the residual force of the levator complex (levator palpebrae superioris muscle/Müller's muscle) is enhanced, leading to eyelid elevation. In cases of ptosis secondary to botulinum toxin use, it is difficult to quantify the extent of involvement of the levator palpebrae superioris or Müller's muscle; however, it is believed that the latter plays a greater role in recovery [19, 20].

This technique should not be considered a first-line treatment, as it is recommended only for experienced injectors. Incorrect application can lead to adverse events such as lagophthalmos or even worsening of ptosis if the toxin spreads to the levator complex [2]. It is recommended for moderate-to-severe ptosis cases refractory to other therapeutic options and as a last resort for mild ptosis cases with significant psychological and functional impacts on the patient [19].

The transdermal injection technique into the pretarsal portion of the orbicularis oculi muscle involves using a solution of 100 units of botulinum toxin reconstituted in 2 mL of saline solution. The patient should look straight ahead, and the following steps should be performed [19, 21]:

1. Inject 2 mm above the lash line of the upper eyelid at the medial iris line.
2. Inject 2 mm above the lash line at the lateral iris line.
3. Inject 2 mm above the lash line, 0.5 cm lateral to the lateral iris line.

**Figure 4:**
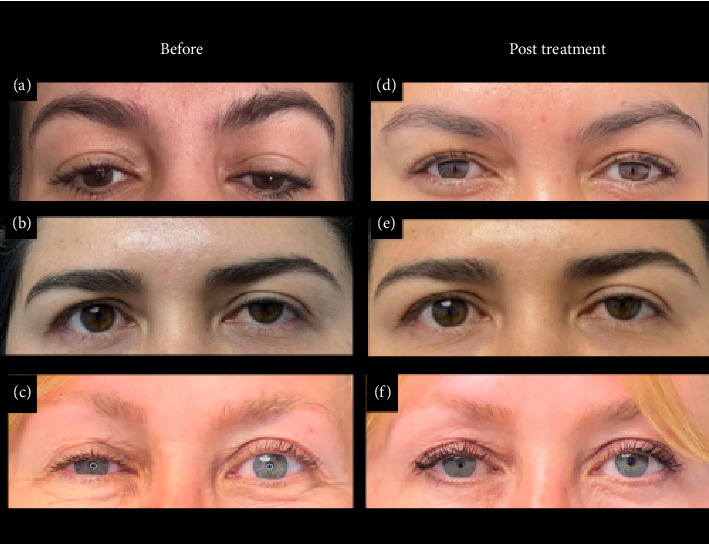
(a) Mild pretreatment eyelid ptosis. (b) Moderate pretreatment eyelid ptosis. (c) Severe pretreatment eyelid ptosis. (d–f) Posttreatment results after botulinum toxin application using the pretarsal technique.

Through this publication, we present three extra institutional cases referred for the management of mild-to-moderate iatrogenic blepharoptosis with pretarsal botulinum toxin. These patients had not responded to previous treatments but achieved satisfactory esthetic results under the care of an expert injector in complications (Figure 4). In addition to esthetic improvement, the patients reported relief from the sensation of ocular heaviness that limited their visual field.

This case series highlights the use of BoNT-A in moderate and mild ptosis cases with minimal response to other treatments. Although it is a highly complex technique, it can provide significant relief for patients who have experienced this type of complication.

Through this article, we highlight the importance of a thorough understanding of anatomy by injectors, present the pretarsal botulinum toxin application technique for managing iatrogenic blepharoptosis caused by BoNT-A, and emphasize the necessity of adequate training in handling complications. In addition, we propose a treatment algorithm based on the severity of eyelid ptosis according to the marginal reflex distance.

A well-trained physician can minimize the consequences of an adverse event by acting promptly and employing algorithms and a methodical approach to treatment [17].

**Figure 5:**
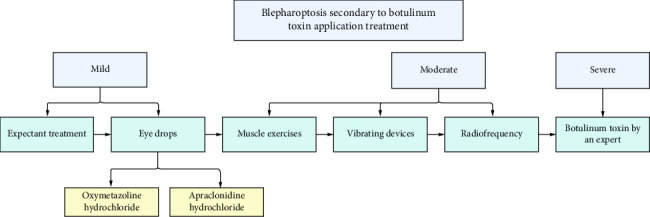
Blepharoptosis secondary to botulinum toxin application treatment algorithm.

In the following, we present our therapeutic algorithm for secondary blepharoptosis due to botulinum toxin application (Figure 5), broken down by severity into mild, moderate, and severe categories. However, it is important to note that most cases require a multimodal approach, combining various therapeutic strategies to achieve the greatest possible reversibility of eyelid opening [19, 22].

In mild cases, expectant management is considered, along with the use of eye drops such as oxymetazoline hydrochloride, 0.5% apraclonidine hydrochloride, or 0.15% brimonidine tartrate (one drop three times a day). These drops can directly stimulate the sympathetic innervation of Müller's muscle, potentially lifting the eyelid by 1–2 mm [19]. In addition, muscle exercises and the use of vibratory devices are recommended. In refractory cases, strategies such as radiofrequency may be added, and as a last resort, pretarsal botulinum toxin application should be performed exclusively by an expert injector due to the high risk of exacerbating eyelid ptosis [23, 24].

In moderate cases, the above strategies are included, with the addition of radiofrequency and pretarsal botulinum toxin application [23, 24].

Finally, in severe cases, pretarsal botulinum toxin application is considered, to be performed by an expert injector with experience in managing complications [23, 24].

## 4. Conclusion

Blepharoptosis following esthetic treatment with BoNT-A is a relatively uncommon complication in trained injectors, yet it has a significant esthetic and functional impact on patients. Therefore, it is important to be familiar with the treatment options for this complication, including the use of neurotoxin via the pretarsal injection technique, which should only be performed by injectors with extensive training and experience.

## Figures and Tables

**Table 1:** The severity scale for eyelid ptosis.

Severity grade	Measurement in millimeters (mm)
Mild	1-2
Moderate	3-4
Severe	> 4

## Data Availability

Data sharing is not applicable to this article as no datasets were generated or analyzed during the current study.
